# Baicalin mitigates cognitive impairment and protects neurons from microglia‐mediated neuroinflammation via suppressing NLRP3 inflammasomes and TLR4/NF‐κB signaling pathway

**DOI:** 10.1111/cns.13086

**Published:** 2019-01-24

**Authors:** Xin Jin, Ming‐Yan Liu, Dong‐Fang Zhang, Xin Zhong, Ke Du, Ping Qian, Wei‐Fan Yao, Hua Gao, Min‐Jie Wei

**Affiliations:** ^1^ Department of Pharmacognosy School of Pharmacy China Medical University Shenyang China; ^2^ Department of Pharmacology School of Pharmacy China Medical University Shenyang China; ^3^ Division of Pharmacology Laboratory National Institutes for Food and Drug Control Beijing China; ^4^ Liaoning Key Laboratory of Molecular Targeted Anti‐Tumor Drug Development and Evaluation Shenyang China

**Keywords:** Alzheimer's disease, baicalin, cognitive impairment, neuroinflammation, neuronal protection

## Abstract

**Aims:**

Baicalin (BAI), a flavonoid compound isolated from the root of *Scutellaria baicalensis* Georgi, has been established to have potent anti‐inflammation and neuroprotective properties; however, its effects during Alzheimer's disease (AD) treatment have not been well studied. This study aimed to investigate the effects of BAI pretreatment on cognitive impairment and neuronal protection against microglia‐induced neuroinflammation and to explore the mechanisms underlying its anti‐inflammation effects.

**Methods:**

To determine whether BAI plays a positive role in ameliorating the memory and cognition deficits in APP (amyloid beta precursor protein)/PS1 (presenilin‐1) mice, behavioral experiments were conducted. We assessed the effects of BAI on microglial activation, the production of proinflammatory cytokines, and neuroinflammation‐mediated neuron apoptosis in vivo and in vitro using Western blot, RT‐PCR, ELISA, immunohistochemistry, and immunofluorescence. Finally, to elucidate the anti‐inflammation mechanisms underlying the effects of BAI, the protein expression of NLRP3 inflammasomes and the expression of proteins involved in the TLR4/NF‐κB signaling pathway were measured using Western blot and immunofluorescence.

**Results:**

The results indicated that BAI treatment attenuated spatial memory dysfunction in APP/PS1 mice, as assessed by the passive avoidance test and the Morris water maze test. Additionally, BAI administration effectively decreased the number of activated microglia and proinflammatory cytokines, as well as neuroinflammation‐mediated neuron apoptosis, in APP/PS1 mice and LPS (lipopolysaccharides)/Aβ‐stimulated BV2 microglial cells. Lastly, the molecular mechanistic study revealed that BAI inhibited microglia‐induced neuroinflammation via suppression of the activation of NLRP3 inflammasomes and the TLR4/NF‐κB signaling pathway.

**Conclusion:**

Overall, the results of the present study indicated that BAI is a promising neuroprotective compound for use in the prevention and treatment of microglia‐mediated neuroinflammation during AD progression.

## 1 INTRODUCTION

Neuroinflammation is closely related to neuronal apoptosis during the progression of Alzheimer's disease (AD).^1^ The pathological hallmarks of neuroinflammation seen in AD are due to the presence of activated microglia within β‐amyloid (Aβ) deposits.^2^ Microglial cells, the resident macrophages in the CNS parenchyma, play an important role in neuroinflammatory and immune responses.^3^ They serve as the first line of defense in the brain and protect the CNS from insults. Microglial cells are promptly activated in response to a variety of stimuli, including infectious, pathological stimuli or Aβ peptide. Activated microglia are able to secrete vast amounts of proinflammatory cytokines, such as tumor necrosis factor (TNF)‐α and interleukin (IL)‐1β, which generate neuroinflammation and cause neuronal apoptosis or death, eventually resulting in the behavioral and psychological symptoms of AD.^4^, ^5^ Therefore, based on the neuropathological features of the AD etiology, the inhibition of microglial activation and the protection of neurons from neuroinflammation may be an alternative strategy that could be used for the treatment of AD.

Currently, the accumulated epidemiological evidence has demonstrated that conventional nonsteroidal anti‐inflammation drug (NSAID) therapies are, to a certain extent, able to reduce neuroinflammatory responses, delay AD progression, and reduce the severity of cognitive impairment.^6^ Regrettably, the incidence of gastrointestinal complications limits the long‐term clinical use of NSAIDs for primary treatment of AD.^7^ Consequently, the development of a drug with either limited or no side effects that can inhibit microglial activation and neuroinflammation has become necessary for the treatment of AD. Interestingly, natural products with fewer side effects and safer properties have been widely consumed or used as therapeutic agents by human societies for many centuries.^8^ Recently, several studies have reported that a great number of natural products and their derivatives have been developed into new or already approved drugs for the treatment of diseases that play a vital role in supporting drug discovery and development.^9^ Moreover, natural products have been shown to have valuable effects during the treatment of microglia‐mediated neuroinflammation in numerous studies.^10^, ^11^ Therefore, they can be considered as an alternative strategy that can be used in the discovery of potential therapeutic drugs for treating AD.

Baicalin (BAI), a flavonoid compound, is responsible for most of the biological effects of *Scutellaria baicalensis*, which has been traditionally used as an herbal medicine in China for many centuries to treat various inflammatory diseases. The anti‐inflammation potential of BAI has been observed in many modern experimental models of both peripheral and central inflammation.^12^, ^13^ Recently, the available evidence has suggested that BAI may exert neuroprotective effects in neurodegenerative disease‐related models. Studies have shown that BAI inhibited apoptosis of Aβ‐induced SH‐SY5Y cells, attenuated oxygen‐glucose deprivation–induced microglial activation, and improved chronic corticosterone‐induced cognitive impairment.^14^, ^15^, ^16^ In addition, BAI also reversed impairment of synaptogenesis and memory deficits in rats affected by minimal hepatic encephalopathy (MHE).^17^ These findings indicated that BAI may represent a promising candidate that could be used for neurodegeneration treatment and reduction of the risk of AD. However, it is unknown whether BAI beneficially affects cognitive function and attenuates inflammatory reactions in APP/PS1 mice.

Therefore, the goal of this study was to evaluate the effect of chronic BAI administration on behavior/cognition, microglial activation, neuronal apoptosis, and neuroinflammatory responses, as well as potential anti‐inflammation mechanisms, in both APP/PS1 mice and LPS/Aβ‐induced BV2 cells.

## 2 MATERIALS AND METHODS

### 2.1 Chemicals

BAI (95% purity; Aladdin Chemicals Ltd., Shanghai, China) was suspended in 0.3% sodium carboxyl methyl cellulose (CMC‐Na) aqueous solution at a concentration of 10.3 mg/mL. Aβ_1‐42_ (Sigma‐Aldrich, St. Louis, USA) was diluted in sterile PBS at a final concentration of 1 mg/mL. The solution was then allowed to aggregate for 7 days at 37°C.

### 2.2 Cell culture

The immortalized murine microglial cell line BV2 and human neuroblastoma SH‐SY5Y cells were purchased from the Chinese Academy of Medical Sciences (Beijing, China). BV2 cells were cultured in high‐glucose DMEM containing 5% fetal bovine serum (HyClone Inc., UT, USA) at 37°C with 5% CO_2_, and 100 U/mL of penicillin/streptomycin. SH‐SY5Y cells were cultured in DMEM/F‐12 containing with 10% fetal bovine serum (HyClone Inc) at 37°C with 5% CO_2_, and 100 U/mL of penicillin/streptomycin.

### 2.3 CCK‐8 cell viability assay

The cell viability was measured using CCK‐8 kit according to the manufacturer's instructions (Dojindo, Kumamoto, Japan). Briefly, BV2 cells were plated in 96‐well plates at a density of 4 × 10^3^ cells/well. Subsequently, the cells were treated with different concentrations of BAI (0, 10, 20, and 40 μmol/L) for 72 hours. The cells were pretreated with different concentrations of BAI (0, 10, 20, and 40 μmol/L) for 1 hour followed by the stimulation with 10 μmol/L Aβ_1‐42_ and 1 μg/mL LPS for 24 hours. After incubation, the CCK‐8 solution (10 μL/well) was added to each well. Then, the cells were incubated for 3 hours at 37°C. The optical density was determined at 450 nm using a microplate spectrophotometer (Multiskan FC; Thermo Fisher Scientific Inc., MA, USA). The cell viability was expressed as the percentage of Abs 450 nm of vehicle control. All assays were performed in triplicate.

### 2.4 Microglia‐conditioned media system

A microglia‐conditioned media system was used as previously described.^18^ BV2 cells were plated in 6‐well culture plates at a density of 2 × 10^5^ cells/well. After overnight incubation, the cells were pretreated with different concentrations of BAI for 1 hour followed by stimulation with 10 μmol/L Aβ_1‐42_ and 1 μg/mL LPS for 24 hours. Following treatment, the supernatant was removed, and the BV2 cells were washed and incubated with fresh medium without any drug for another 24 hours. The conditioned media (CM) from wells containing only microglia, wells containing LPS/Aβ_1‐42_ (CM‐LPS/Aβ), and wells subject to treatment with BAI (CM‐LPS/Aβ + BAI) were collected and applied to SH‐SY5Y cells that had been seeded in 96‐well culture plates at a density of 2 × 10^3^ cells/well for 24 hours. The proliferation of SH‐SY5Y cells was measured using a CCK‐8 assay and flow cytometry (MACSQuant VYB; Miltenyi Biotec Technology Co., Germany) according to the instructions included in the Annexin V‐FITC Kit (Sungene Biotechnology Co., Tianjin, China).

### 2.5 Animals and drug administration

APP/PS1 transgenic mice were purchased from the Jackson Laboratory (*B6C3‐Tg [APPswe, PSEN1dE9] 85Dbo/J*). The transgenic offspring that overexpressed APP/PS1 were compared to their wild‐type (WT) littermates, according to age and C57Bl/6 background. The offspring were genotyped using PCR with tail‐tip DNA. The specific primer sequences were as follows: APP (forward: AGGACTGACCACTCGACCAG; reverse: CGGGGGTCTAGTTCTGCAT); and PS1 (forward: AATAGAGAACGGCAGGAGCA; reverse: GCCATGAGGGCACTAATCAT). The PCR cycling conditions consisted of 40 cycles of denaturation at 98°C for 10 seconds, annealing at 60°C for 15 seconds, and extension at 68°C for 1 minutes The PCR products were separated on 1.5% agarose gels and then visualized using GelRed (1:10000; BBI Co. Ltd., Shanghai, China). The male animals (22‐31 g, 14 months) were housed 3‐5 per cage in a facility with a 12‐hour light/dark cycle (lights on from 7:00 a.m. to 7:00 p.m.), 55% relative humidity, and a temperature of 22 ± 1°C, and water and food were available ad libitum. The mice were allowed to adapt to the laboratory conditions before testing. All experimental procedures and animal care were conducted according to the Standard Medical Laboratory Animals Care and Use Protocols (Ministry of Health PR China, 1998) and the Laboratory Animal Ethical Standards of China Medical University.

Twenty APP/PS1 transgenic mice were randomly and equally assigned into two groups (n = 10): the BAI‐treated APP/PS1 group (BAI group) and the vehicle‐treated APP/PS1 group (APP/PS1 group). Ten wild‐type littermates (WT group) were assigned to the control group. A previous study confirmed that the administration of BAI at a dose of 100 mg/kg intraperitoneally (*i.p*.) to C57BL/6 mice did not induce the production of inflammatory cytokines and inflammation in the brain.^19^ Thus, a group of BAI‐treated littermates (WT‐BAI group) was not established. The mice in the BAI group were intragastrically (*i.g*.) administered 103 mg/kg BAI. The APP/PS1 and WT groups were *i.g*. administered a 0.3% CMC‐Na aqueous solution instead of BAI. All mice were treated once a day for 33 days.

### 2.6 Passive avoidance test

The Passive avoidance test (PAT) protocol that was used was based on previous studies.^20^ The apparatus (BA‐200; Taimeng Tech. Co. Ltd., Chengdu, China) was divided into two compartments, an illuminated compartment and a dark compartment, that were connected together via an automatic guillotine door. The dark compartment was equipped with a grid floor through which a footshock could be delivered. Mice underwent two independent trials: a training session and a test session that was conducted 24 hours later. In the training session, the mice were initially placed into the light compartment, and after 3 minutes of habituation, the guillotine door was opened to allow the mice to enter the dark compartment. The door was closed as soon as the mice passed into the dark compartment, and a mild electrical footshock (0.5 mA, 2 seconds) was delivered. After 10 seconds, the mice were removed from the dark compartment and returned to their cages. The test session was performed 24 hours later, during which each mouse was reevaluated in a similar manner. However, a footshock was not delivered, and the latencies and frequencies for the mice in switching from the light compartment to the dark compartment were recorded up to a maximum of 300 seconds.

### 2.7 Morris water maze test

The Morris water maze test (MWM) protocol used in this study was obtained from a previous report.^21^ The MWM was conducted in a circular water pool (120 cm in diameter; 50‐cm‐high wall) filled with water maintained at 23 ± 1°C. An invisible fixed platform (10 cm diameter) was immersed 1 cm below the water surface. On the baseline day (day 0), all mice underwent free swimming for 1 minute in the pool without the presence of the platform, in order to familiarize them with the apparatus. On training days 1‐5, the mice were subject to 60 seconds of spatial acquisition during four trials per day, with a 15‐minute intertrial interval, during which they were gently released into the water from the pool rim. The trial automatically terminated once the mice found and climbed onto the platform. Different starting positions were used for each trial. If a mouse did not reach the platform within 60 seconds, it was gently guided back to the platform for the remaining 60 seconds. During each trial, the escape latency and the path length used by the mice to find the hidden platform were recorded to analyze the performance of mice. A probe trial was conducted on day 6 to assess memory consolidation, during which the platform was removed from the maze, and the animals were allowed to swim freely for 60 seconds. The number of times that each mouse crossed over the former platform location and the percentage of time spent exploring in the quadrant were monitored and recorded. All the data were obtained using a video tracking system (MWT‐100; Taimeng Tech. Co. Ltd., Chengdu, China).

### 2.8 Locomotivity test

After the PAT and MWM were conducted, a locomotivity test was conducted to evaluate the motor function of mice using a locomotivity testing paradigm system (ZZ‐6; Taimeng Tech. Co. Ltd., Chengdu, China). Briefly, the mice were placed into the system and allowed to explore for 10 minutes. The cages were routinely cleaned with 75% ethanol following each session. The parameters of locomotivity and stand‐up for each mouse were recorded.

### 2.9 Tissue collection

After completing the behavioral tests, the mice were killed by decapitation, and the brains were rapidly removed and cut sagittally into left and right hemispheres on an ice‐cooled board. The left hemi‐brain was immediately fixed in 4% paraformaldehyde for 48 hours at 4°C for the purposes of immunohistochemistry and immunofluorescence. The hippocampus and cerebral cortex of the right hemi‐brain were dissected and removed. The isolated hippocampal tissues were carefully minced into small pieces that were approximately 0.5 mm thick and were then homogenized in RIPA buffer (Beyotime Biotechnology, Beijing, China) containing 0.1% protease inhibitor PMSF (Sigma‐Aldrich) and TRIzol agent (Beyotime Biotechnology, Beijing, China) for subsequent protein and RNA isolation, respectively. The tissue samples were frozen at −80°C for further biochemical analysis.

### 2.10 Immunohistochemistry and immunofluorescence

Immunohistochemistry was performed as previously described.^22^ Briefly, paraffin‐embedded slices (4 μm) were quenched to inactivate endogenous peroxidase for 30 minutes using 0.5% H_2_O_2_; then, the nonspecific binding sites were blocked using 5% BSA (bovine serum albumin) at room temperature for 1 hour. The sections were incubated overnight at 4°C with Cleaved‐caspase‐3 (CASP3) antibody (1:200; CST, MA, USA). After washing, sections were incubated for 1 hour in the appropriate secondary antibody at 37°C. The immunoreactivity was visualized using a DAB kit (Beyotime Biotechnology, Beijing, China). A light microscope (ECLIPSE E600; Nikon, Tokyo, Japan) was used to observe the sections, and the intensity of the stained areas for each group was analyzed using Image‐Pro Plus 6.0 software (Media Cybernetics, MD, USA).

Immunofluorescence microscopy was performed on brain and cell slices as previously described.^23^ The slices were blocked with 5% BSA in PBS for 30 minutes at room temperature followed by an overnight incubation at 4°C with goat anti‐Iba‐1 (1:500; Wako, Osaka, Japan), rabbit anti‐Aβ_1‐40_ antibody (1:800; CST), rabbit anti‐TLR4 (1:300; Abcam, Cambridge, UK), and rabbit anti‐NLRP3 (1:500; CST) diluted in PBS. After primary antibody incubation, the slices were extensively washed with PBS and then incubated with Alexa 488‐ or Alexa 594‐conjugated IgG secondary antibodies (1:200; Invitrogen, MA, USA) for 1 hour at 37°C in the dark, and then were rinsed three times with PBS. Nuclear staining was performed using 1 μg/mL 4′,6‐diamidino‐2‐phenylindole (DAPI; Sigma‐Aldrich) for 10 minutes followed by exhaustive washing in distilled water. The slices were subsequently examined using a confocal laser scanning microscope (C2; Nikon, Tokyo, Japan). The Aβ plaques were assessed by measuring the fluorescence intensity of the Aβ‐positive staining in the center region of the hippocampus of each mouse (n = 8/group, 3 sections/brain). The total number of Iba‐1‐positive cells surrounding the Aβ plaques in the hippocampal center within a 0.7 × 0.7 square millimeter of each section was counted manually, and the mean ± SD per square millimeter was determined for each brain. The cells defined as Iba‐1‐positive were required to simultaneously satisfy the following criteria: (i) the presence of red Iba‐1 staining in the cell body; and (ii) the possible center location of the cells clearly having blue nuclear DAPI staining. Areas of slight Iba‐1‐staining with no clear cell structure characteristics were classified as unclear background and were not included in the analysis. The images were analyzed using Image‐Pro Plus 6.0 software (Media Cybernetics).

### 2.11 Hoechst 33258 staining

Briefly, the brain tissue slices were dewaxed and dehydrated, and subsequently stained with Hoechst 33258 solution (Wanleibio Co. Ltd., Shenyang, China) for 30 minutes in the dark. The staining solution was then removed, and the slices were washed three times with PBS. The images of stained nuclei were captured using a fluorescence microscope (C2; Nikon, Tokyo, Japan). The cells observed within the slides were considered apoptotic if the nuclei presented chromatin condensation and/or fragmentation (brightly stained). The numbers of normal and apoptotic nuclei in randomly selected regions were counted. The percentage of Hoechst‐positive cells was then quantified.

### 2.12 Real‐time RT‐PCR

The RNA concentration was determined using a spectrophotometer (NanoDrop 2000; Thermo Fisher Scientific Inc.). cDNA was synthesized from total RNA using the ReverTra Ace qPCR RT kit (Toyobo Co., Ltd, Osaka, Japan). The cDNA was diluted 10‐fold prior to being used for RT‐PCR. Quantitative analysis of the expression of IL‐1β, IL‐18, iNOS, and GAPDH mRNA was performed using RT‐PCR with 96‐well optical reaction plates using the Mx3000P qPCR System (Agilent Technologies Inc., CA, USA). RT‐PCR was performed using the following conditions: initial denaturation at 95°C for 30 seconds, followed by 40 cycles of denaturation at 95°C for 5 seconds and annealing and extension at 60°C for 30 seconds.

The primer sequences that were used are presented in Table 1. The primers used in the present study were selected from the PubMed database and synthesized by Shanghai Sangon Biological Engineering Co., Ltd. The RT‐PCR data were analyzed using the relative gene expression changes (2^−ΔΔCt^) method. The normalization of expression was performed using the housekeeping gene GAPDH. The analysis of each sample was performed in triplicate.

**Table 1:** PCR primer sequences used

Gene target	Forward sequence	Reverse sequence
GAPDH	5′‐AGCCTCGTCCCGTAGACAAAA‐3	5′‐TGGCAACAATCTCCACTTTGC‐3′
IL‐1β	5′‐CCTGCAGCTGGAGAGTGTGGAT‐3′	5′‐TGTGCTCTGCTTGTGAGGTGCT‐3′
IL‐18	5′‐ACGTGTTCCAGGACACAACA‐3′	5′‐GGCGCATGTGTGCTAATCAT‐3′
iNOS	5′‐CCTTGGTGAAGGGACTGAGC‐3′;	5′‐CAACGTTCTCCGTTCTCTTGC‐3′

### 2.13 Western blot

The homogenates isolated from the brain tissues and cells were centrifuged at 18800 g for 15 minutes at 4°C. The protein concentration was measured using a BCA kit (Beyotime Biotechnology, Beijing, China). After heating at 100°C for 10 minutes in loading buffer (Beyotime Biotechnology, Beijing, China), the protein (30 μg per lane) was loaded onto a 12% SDS‐PAGE gel and separated at 120 V for 1 hour, and then transferred onto a PVDF membrane (Millipore, Billerica, MA, USA) at 80 V for 2 hour. The membranes were blocked at room temperature for 1 hour with a 5% BSA solution containing 0.1% Tween‐20 and then incubated with specific primary antibodies overnight at 4°C. Rabbit polyclonal antibodies against Cleaved‐CASP3 (1:1000; CST), NLRP3 (1:1000; CST), Cleaved‐caspase‐1 (CASP1) (1:1000; CST), Iba‐1 (1:500; Wako, Osaka, Japan), IL‐1β (1:1000; CST), IL‐18 (1:1000; Abcam, Cambridge, UK), p‐NF‐κB p65 (1:1000; CST), NF‐κB p65 (1:1000; CST), p‐Iκbα (1:1000; CST), Iκbα (1:1000; CST), and β‐actin (1:1000; CST) were used in this study. After washing seven times with TBST, the membranes were probed with HRP‐conjugated secondary antibodies (1:10000; EarthOx, CA, USA) at room temperature for 1 hour and subsequently visualized using an ECL chemiluminescent kit (ECL Plus; Thermo Scientific, MA, USA). The membranes were analyzed using Quality One analysis software (Bio‐Rad, Hercules, CA, USA).

### 2.14 ELISA measurement

After obtaining brain tissues and cell supernatants, the protein concentrations were measured using a BCA kit (Beyotime Biotechnology, Beijing, China). Total protein was extracted from each sample and subject to quantitative analysis of IL‐18 and IL‐1β levels using an ELISA kit (Chenglin Biotechnology, Beijing, China) according to the manufacturer's instructions. The IL‐18 and IL‐1β levels were measured in duplicate for each sample.

### 2.15 Statistical analysis

All statistical analysis was performed using SPSS 16.0 software. Data were expressed as means ± standard deviation (SD). Statistical differences were analyzed by one‐way ANOVA followed by Fisher's least significant difference (LSD) *t* test. *P* < 0.05 was considered as statistically significant.

## 3 RESULTS

### 3.1 BAI treatment ameliorated learning and memory deficits in APP/PS1 mice

**Figure 1:**
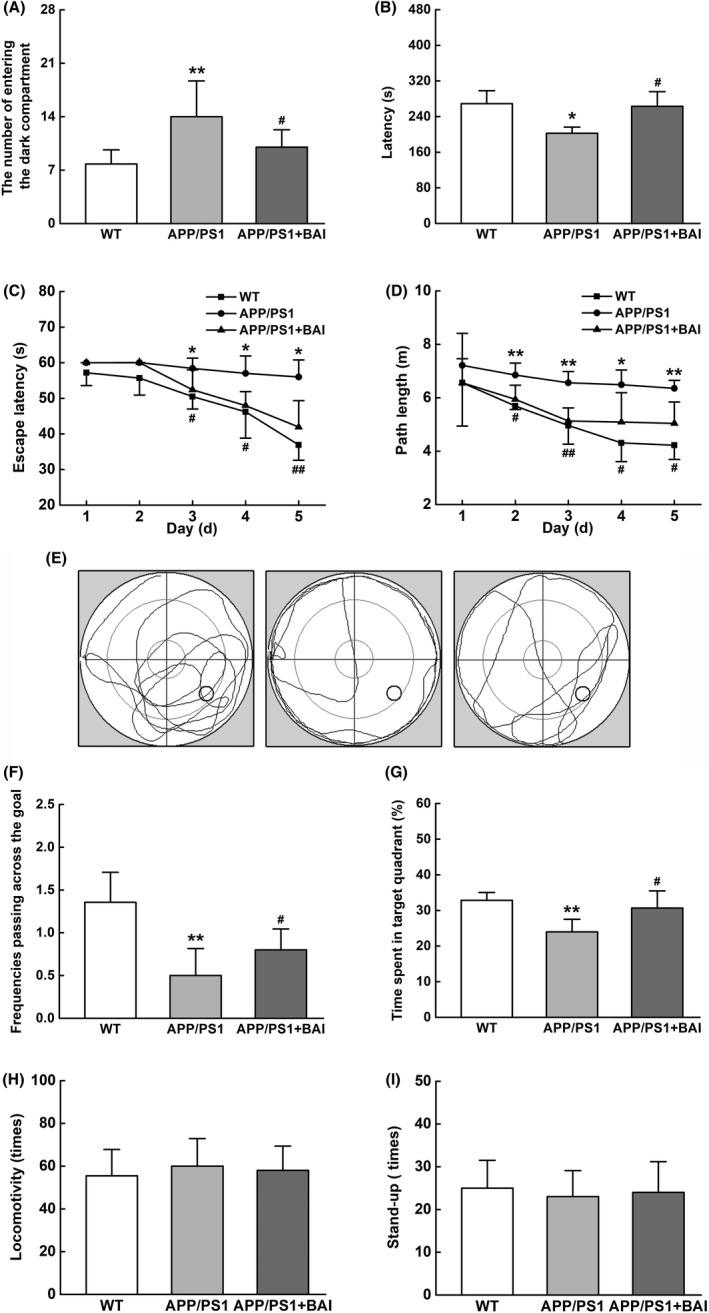
BAI ameliorated learning and memory deficits in APP/PS1 mice. (A, B) The number of entering the dark compartment and the latency of APP/PS1 mice during the passive avoidance test (PAT). (C, D) The escape latency and the path length of APP/PS1 mice in the navigation test of morris water maze test. (E) The representative locus plot in the probe trial. (F, G) The frequency of passing through the goal and the time spent in target quadrant during the probe trial. (H, I) The locomotivity and the frequency of stand‐up in locomotivity test. All data are presented as mean ± SD (n = 10/group). *P < 0.05, **P < 0.01, compared with wild‐type (WT) group; #*P* < 0.05, ##*P* < 0.01, compared with APP/PS1 group

To determine whether BAI treatment ameliorated learning and memory deficits in APP/PS1 mice, behavioral tests were performed to assess the therapeutic effects. After BAI treatment, the mice were subjected to PAT to evaluate their retention of nonspatial memory. The results showed that the APP/PS1 group mice had a higher frequency of entering the dark compartment and a shorter latency when entering the dark compartment than mice of the WT group (Figure 1A, B). Following treatment, however, the frequency of entering the dark compartment in the BAI group was significantly decreased and the latency when entering the dark compartment was clearly prolonged compared with the APP/PS1 group (Figure 1A, B).

After the PAT, the mice were subjected to MWM for consecutive 7 days to evaluate their spatial learning and memory. During navigation testing, there were no significant differences among the WT, APP/PS1, and BAI groups on 1‐2 days. During the following 3‐5 days, the APP/PS1 group had a higher escape latency and longer path length than the WT group (Figure 1C, D), whereas the escape latency and the path length in the BAI group were notably reduced, compared with the APP/PS1 group (Figure 1C, D). Moreover, on the 6th day, the three groups of mice were subjected to a probe trial to evaluate their memory retention. The APP/PS1 group mice had a remarkably decreased swimming time in the target quadrant and mainly swam around in the tank without crossing over the original platform position (Figure 1E). In contrast, the BAI group mice spent more time in target quadrant and had an increased frequency of crossing the platform in comparison with the APP/PS1 group (Figure 1F, G).

The locomotivity and frequencies of stand‐up among the groups were not significantly different (Figure 1H, I). This finding indicated that memory dysfunction in APP/PS1 mice was not affected by motor function in mice before and after BAI treatment. Thus, these data from the behavioral tests demonstrated that BAI treatment ameliorated cognitive deficits in learning and memory functioning in APP/PS1 mice.

### 3.2 BAI did not reduce Aβ deposition, but suppressed microglial activation and proinflammatory cytokine levels in APP/PS1 mice

**Figure 2:**
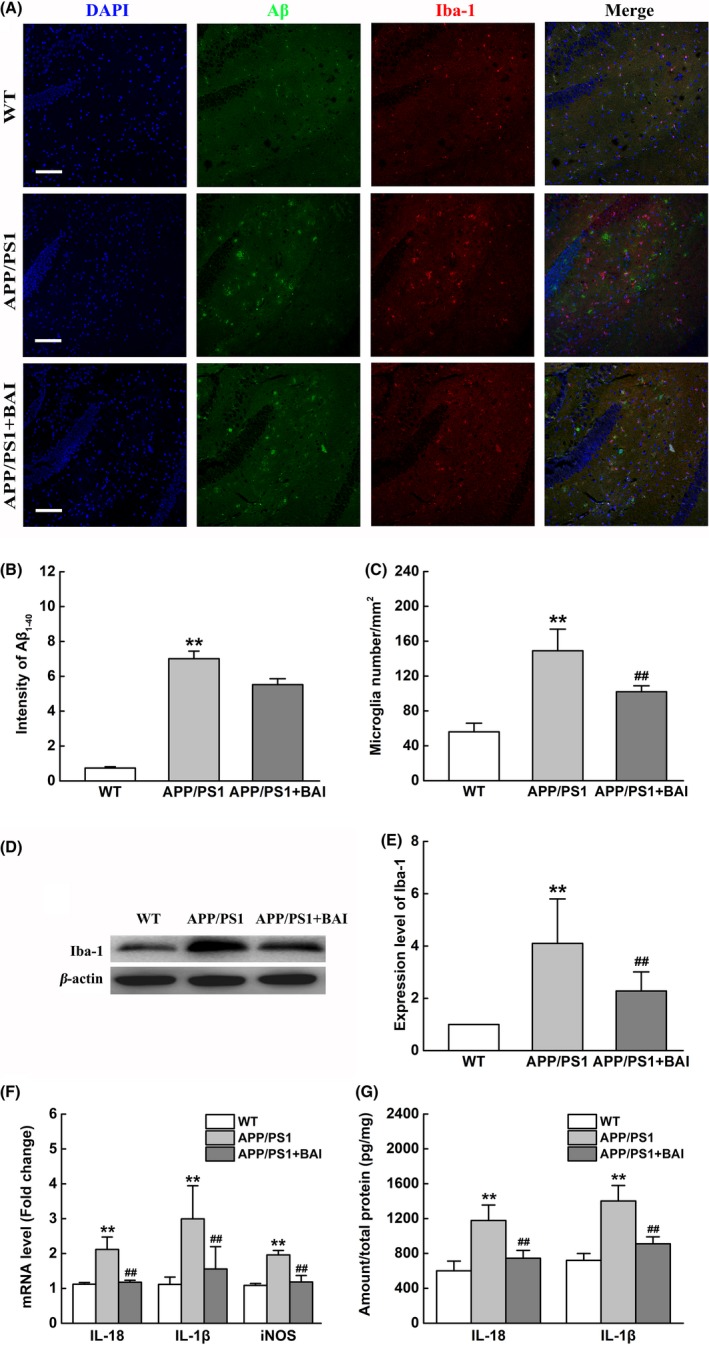
BAI treatment did not reduce Aβ deposition, but decreased microglial activation and proinflammatory cytokine production in APP/PS1 mice. (A) Immunofluorescent images of Aβ_1‐40_ (green)/Iba‐1 (red)/DAPI (blue) colocalization in the hippocampus of APP/PS1 mice. Scale bar: 100 μm. (B) Quantification of immunofluorescent intensity of Aβ_1‐40_ area (n = 8/group). (C) Quantification of microglial immunofluorescent number (n = 8/group). (D) Representative immunoblots probed with antibodies against Iba‐1 and β‐actin. (E) Quantification of the levels of Iba‐1 normalized to β‐actin (n = 4). (F) Graph showing the mRNA levels of IL‐1β, IL‐18, and iNOS in the hippocampus measured using RT‐PCR (n = 4). (G) Graph showing the levels of secreted IL‐1β and IL‐18 in the hippocampus measured using ELISA (n = 4). All data are presented as mean ± SD. **P < 0.01, compared with wild‐type (WT) group; ##*P* < 0.01, compared with APP/PS1 group

An invariant feature of AD brain tissue is microglial activation adjacent to Aβ deposits.^24^ We evaluated the effects of BAI treatment on Aβ plaques and microglial activation in the hippocampus of APP/PS1 mice using immunofluorescence. The results showed that no Aβ plaques were observed in the WT group. However, the APP/PS1 group showed many Aβ_1‐40_‐positive plaques that were distributed throughout the hippocampus. After BAI treatment, the plaque number and total area of deposition of Aβ_1‐40_ in mice in the BAI group were slightly reduced compared with mice in the APP/PS1 group, but the difference was not statistically significant (Figure 2A, B).

We then analyzed the correlation between the number of Iba‐1‐positive cells and the Aβ plaque area among the different groups. Immunofluorescence results demonstrated a stronger presence of Iba‐1‐positive microglia in the area surrounding Aβ deposits within mice in the APP/PS1 group, whereas BAI treatment reduced the number of activated microglia (Figure 2A, C). Meanwhile, we measured Iba‐1 levels using Western blot assays. The results confirmed these observations by showing that the Iba‐1 expression levels in the BAI group were markedly reduced compared to those of the APP/PS1 group (Figure 2D, E).

Furthermore, we investigated whether BAI reduced the levels of proinflammatory cytokines in the hippocampus of APP/PS1 mice. RT‐PCR results showed that the mRNA levels of IL‐1β, IL‐18, and iNOS were remarkably upregulated in the APP/PS1 group, and BAI treatment significantly reduced the mRNA levels of IL‐1β, IL‐18, and iNOS (Figure 2F). To confirm the above results, ELISA was performed, which showed that the levels of IL‐1β and IL‐18 were remarkably increased in the hippocampus of the APP/PS1 group mice. However, BAI treatment significantly reduced the levels of IL‐1β and IL‐18, compared to that in the APP/PS1 mice (Figure 2G). Overall, these data demonstrated that BAI administration did not affect Aβ plaque maturation, but did attenuate microglial activation and neuroinflammation in the hippocampus of APP/PS1 mice.

### 3.3 BAI treatment prevented neuronal apoptosis in APP/PS1 mice

**Figure 3:**
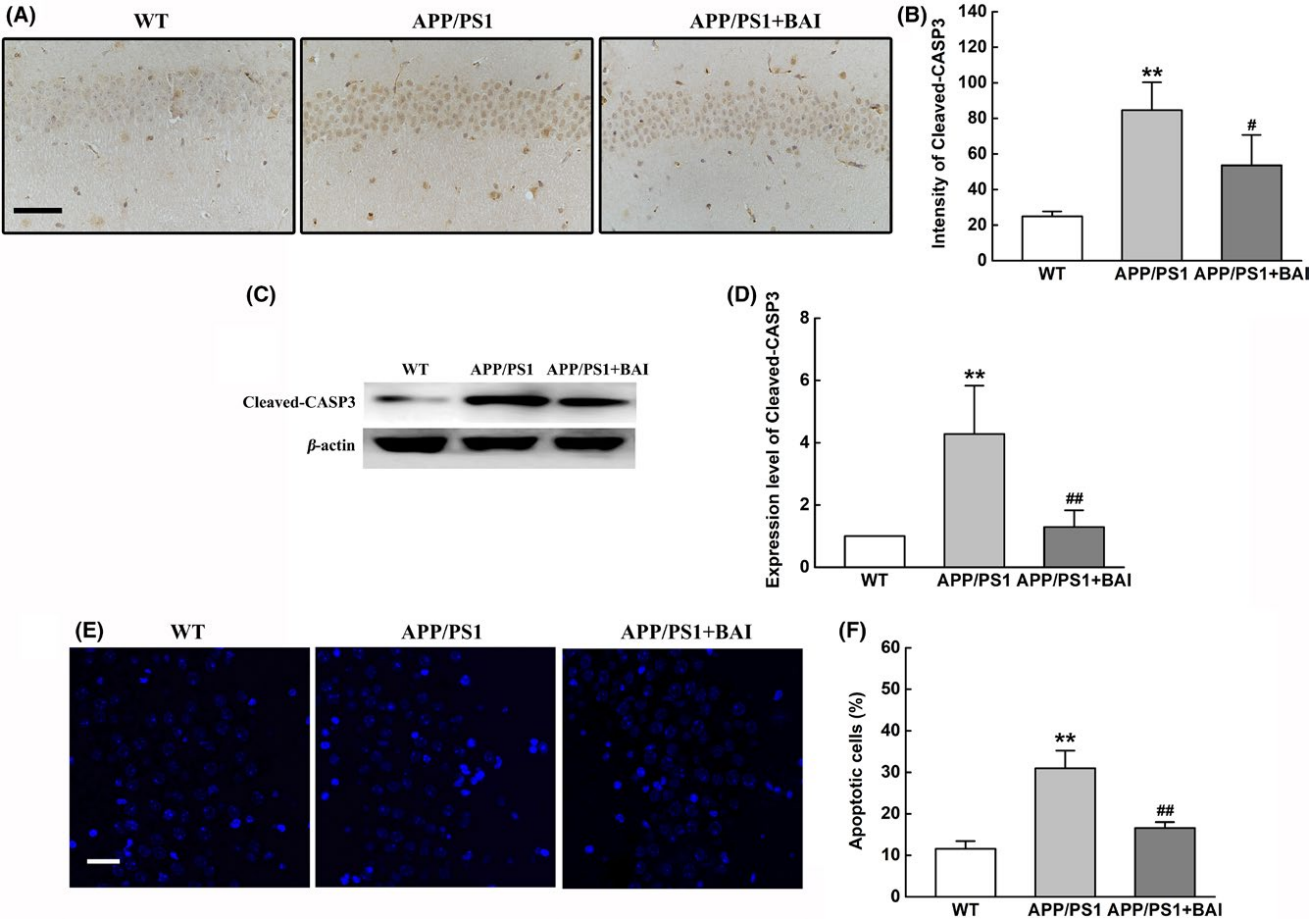
BAI treatments prevented neuronal apoptosis in APP/PS1 mice. (A) Immunohistochemical analysis of Cleaved‐CASP3‐positive cells in the hippocampus of APP/PS1 mice. Scale bar: 50 μm (B) Quantification of Cleaved‐CASP3‐positive cells in the hippocampus of APP/PS1 mice (n = 8/group). (C) Representative immunoblots probed with antibodies against Cleaved‐CASP3 and β‐actin. (D) Quantification of the level of Cleaved‐CASP3 normalized to β‐actin (n = 4). (E) Hoechst 33258 staining of typical apoptotic morphology of condensed and/or fragmented neuronal nuclei (white arrowheads) in the hippocampus of APP/PS1 mice. Scale bar: 100 μm. (F) Quantification of apoptotic cells in the hippocampus of APP/PS1 mice (n = 8/group). All data are presented as mean ± SD. *P < 0.05, **P < 0.01, compared with wild‐type (WT) group; #*P* < 0.05, ##*P* < 0.01, compared with APP/PS1 group

Microglial activation induced by Aβ deposition and the release of overwhelming levels of proinflammatory cytokines causes neuronal apoptosis in AD.^25^, ^26^ To observe whether BAI could attenuate neuronal apoptosis in APP/PS1 mice, we used immunohistochemical and Western blot assays to detect the expression of Cleaved‐CASP3 in the hippocampus. Immunohistochemical staining showed that the number of Cleaved‐CASP3‐positive cells in the hippocampus of mice in the BAI‐treated group was dramatically decreased in comparison with APP/PS1 mice (Figure 3A, B). Western blotting also confirmed that the expression of Cleaved‐CASP3 protein in the hippocampus of mice in the BAI‐treated group was significantly decreased compared to the APP/PS1 group (Figure 3C, D). Consistent with the immunostaining result, Hoechst 33258 staining demonstrated that BAI significantly decreased the percentage of apoptotic nuclei in the hippocampus in APP/PS1 mice (Figure 3E, F). These findings indicated that BAI protected against neuronal apoptosis in the hippocampus of APP/PS1 mice.

### 3.4 BAI inhibited the activation of NLRP3 inflammasomes and the TLR4/NF‐κB signaling pathway in APP/PS1 mice

**Figure 4:**
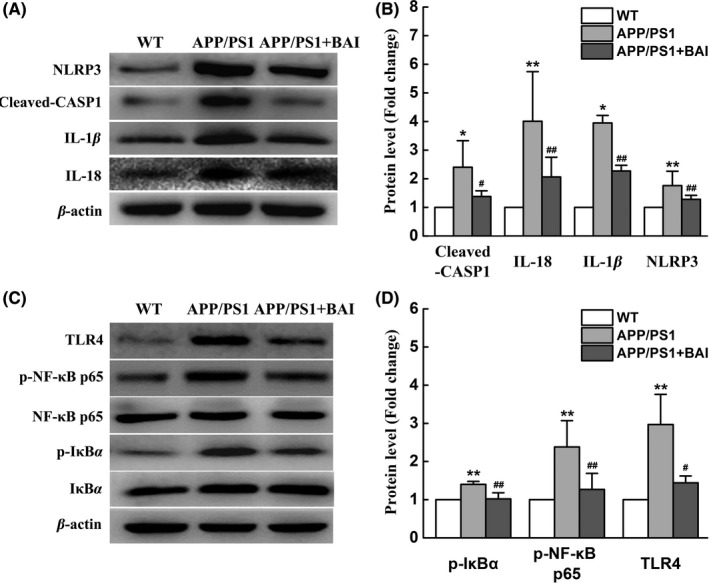
Effects of BAI on the activation of NLRP3 inflammasomes and TLR4/NF‐κB signaling pathway in hippocampal homogenates of APP/PS1 mice. (A) Representative immunoblots probed with antibodies against NLRP3, Cleaved‐CASP1, IL‐1β, IL‐18, and β‐actin. (B) Quantification of the levels of NLRP3, Cleaved‐CASP1, IL‐1β, and IL‐18 normalized to β‐actin. (C) Representative immunoblots probed with antibodies against TLR4, p‐NF‐κB p65, NF‐κB p65, p‐IκBα, IκBα, and β‐actin. (D) Quantification of the levels of TLR4, p‐NF‐κB p65, NF‐κB p65, p‐IκBα, and IκBα normalized to β‐actin. All data are presented as mean ± SD (n = 4). *P < 0.05, **P < 0.01, compared with wild‐type (WT) group; #*P* < 0.05, ##*P* < 0.01, compared with APP/PS1 group

In the present study, it was observed that BAI could reduce the mRNA and protein expression levels of IL‐18 and IL‐1β. Additionally, maturity and secretion of IL‐18 and IL‐1β depend on the activation of NLRP3 inflammasomes. Therefore, one possible mechanism that may underlie proinflammatory cytokine inhibition is suppression of the activation of NLRP3 inflammasomes by BAI. Western blot analysis was performed to measure the activation and expression NLRP3 inflammasomes. The results showed that compared with the WT group, the protein expression of NLRP3 in mice in the APP/PS1 group was significantly increased; however, NLRP3 expression was significantly decreased by BAI. Moreover, Cleaved‐CASP1, IL‐1β, and IL‐18 were upregulated in the APP/PS1 mice compared to the WT mice. Nevertheless, BAI treatment significantly diminished Cleaved‐CASP1, IL‐1β and IL‐18 upregulation in mice in the BAI group compared to mice in the APP/PS1 group (Figure 4A, B). These results indicated that BAI reduced the activation of NLRP3 inflammasomes in APP/PS1 mice.

Stimulation of the NF‐κB signal transduction pathway is thought to be an early event that is essential for NLRP3 inflammasome activation.^27^ To examine the expression of NF‐κB pathway proteins, we conducted Western blot analysis. Our data showed that upon comparison with the control group, p‐IκBα and p‐NF‐κB p65 levels were increased in the APP/PS1 group, suggesting that the NF‐κB signaling pathway was activated. Since NF‐κB can be activated by TLR4, we then measured the relative protein level of TLR4. As expected, mice in the APP/PS1 group showed a significant increase in the level of TLR4, suggesting that the TLR4/NF‐κB signaling pathway had been activated. In contrast, BAI administration downregulated the protein expression of TLR4 and the phosphorylation of IκBα and NF‐κB p65 (Figure 4C, D). Therefore, it was indicated that the activation of TLR4/NF‐κB signaling pathway was mostly likely inhibited by BAI in APP/PS1 mice.

### 3.5 BAI reduced the levels of proinflammatory mediators in LPS/Aβ‐stimulated BV2 cells

**Figure 5:**
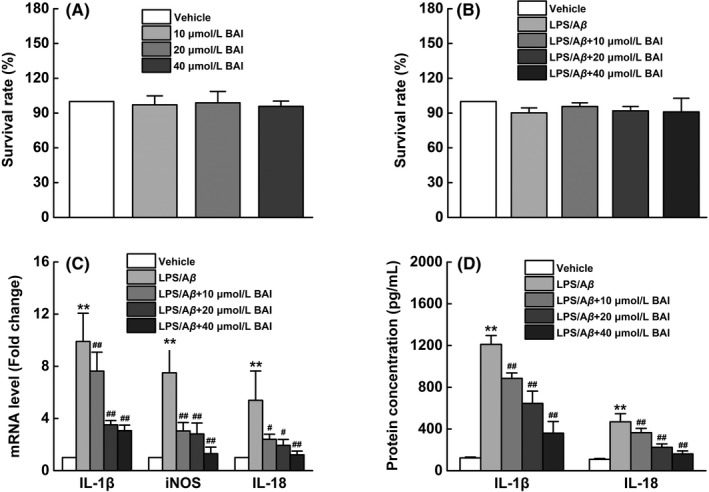
BAI inhibited neuroinflammatory response by the downregulation of the expression of inflammatory mediators in LPS/Aβ‐stimulated BV2 cells. (A) The cell viability assessed using CCK‐8 assay pretreated with or without different concentrations of BAI (10, 20, and 40 μmol/L) for 72 hours in BV2 cells. (B) The cell viability assessed using CCK‐8 assay pretreated with Aβ_1‐42_ (10 μmol/L) and LPS (1 μg/mL) for 1 hours followed by incubation with different concentrations of BAI (10, 20, and 40 μmol/L) for 24 hours in BV2 cells. (C) Graph showing the mRNA levels of IL‐1β, IL‐18, and iNOS analyzed using RT‐PCR. (D) Graph showing the levels of secreted IL‐1β and IL‐18 analyzed using ELISA. All data are presented as mean ± SD (n = 4). *P < 0.05, **P < 0.01, compared with vehicle group; #*P* < 0.05, ##*P* < 0.01, compared with LPS/Aβ‐treated group

To further investigate the role of BAI in microglia‐mediated inflammation, BV2 microglial cell lines were cultured and exposed to LPS/Aβ as indicated. We first evaluated the cytotoxicity of BAI (10, 20, and 40 μmol/L) in BV2 microglial cells. The CCK‐8 assay results showed that BAI at a concentration of <40 μmol/L induced no toxicity in BV2 cells (Figure 5A). Next, we tested the cytotoxicity of BAI (10, 20, and 40 μmol/L) when it was incubated with LPS/Aβ and found that this also produced no cytotoxicity in BV2 cells at all tested concentrations (Figure 5B). We further examined the effects of BAI on IL‐1β, IL‐18, and iNOS mRNA and protein levels in LPS/Aβ_1‐42_‐induced BV2 cells using RT‐PCR and ELISA. The results showed that BAI treatment significantly inhibited LPS/Aβ‐induced mRNA expression of IL‐1β, IL‐18, and iNOS (Figure 5C), as well as levels of secreted IL‐1β and IL‐18, in a concentration‐dependent manner (Figure 5D). Collectively, these findings suggested that BAI could exert anti‐inflammation effects in LPS/Aβ‐stimulated BV2 cells by inhibiting the production of proinflammatory mediators. As a result, a 40 μmol/L BAI concentration was selected for use in future experiments.

### 3.6 BAI inhibited microglial activation and morphological changes induced by LPS/Aβ‐induced BV2 cells

**Figure 6:**
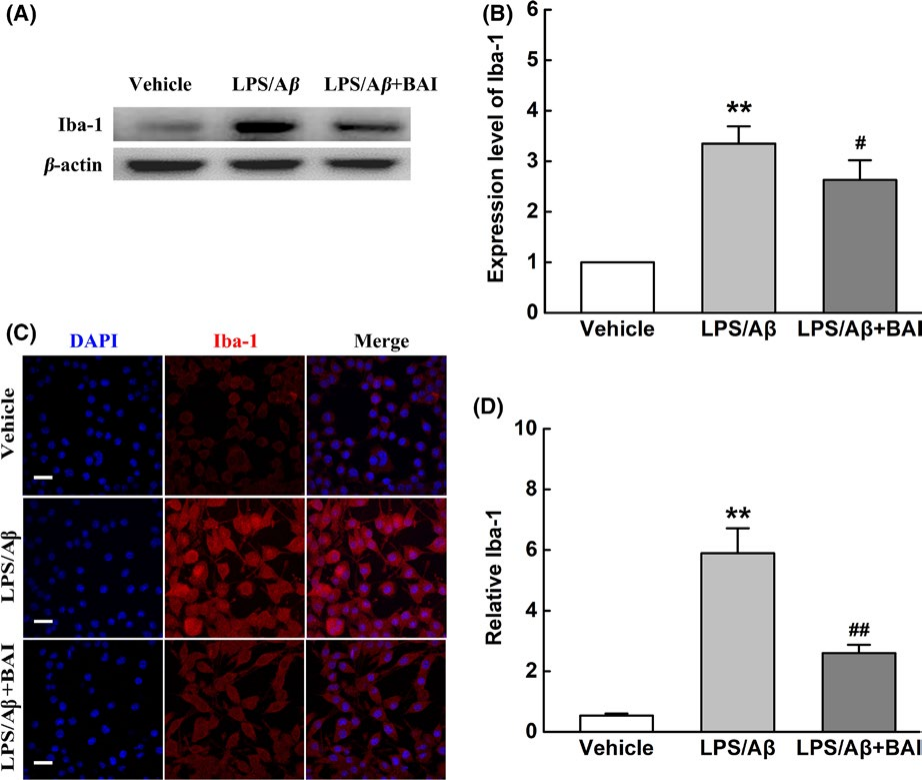
BAI treatment inhibited microglial activation and morphological changes induced by LPS/Aβ‐induced BV2 cells. (A) Representative immunoblots probed with antibodies against Iba‐1 and β‐actin. (B) Quantification of the level of Iba‐1 normalized to β‐actin. (C) Fluorescent Iba‐1 (red)/DAPI (blue) immunostaining in LPS/Aβ‐induced BV2 cells. Scale bar: 30 μm. (D) Quantification of Iba‐1 immunofluorescence. All data are presented as mean ± SD (n = 4). **P < 0.01, compared with vehicle group; #*P* < 0.05, ##*P* < 0.01, compared with LPS/Aβ‐treated group

Activated microglia are important mediators of Aβ‐induced neurotoxicity via the production of proinflammatory cytokines.^28^ To further evaluate whether BAI suppressed microglial activation in LPS/Aβ‐stimulated BV2 cells, we analyzed Iba‐1 protein expression levels using Western blot and immunofluorescence. The results showed that LPS/Aβ stimulation triggered microglial activation and increased the expression of Iba‐1. After BAI treatment, Iba‐1 protein levels showed a prominent decrease (Figure 6A, B). Moreover, the analysis of cell morphology clearly showed that the cell bodies of normal microglia were small and round and had few processes; however, LPS/Aβ‐activated cells were larger and had an amoeboid morphology with retraction of extensions. In addition, BAI treatment could markedly reverse these morphological changes in microglia (Figure 6C, D). These results suggest that the pretreatment effects of BAI in AD were closely related to its inhibition of microglial activation and morphological changes.

### 3.7 BAI reduced microglial neurotoxicity and protected neural cells in vitro

**Figure 7:**
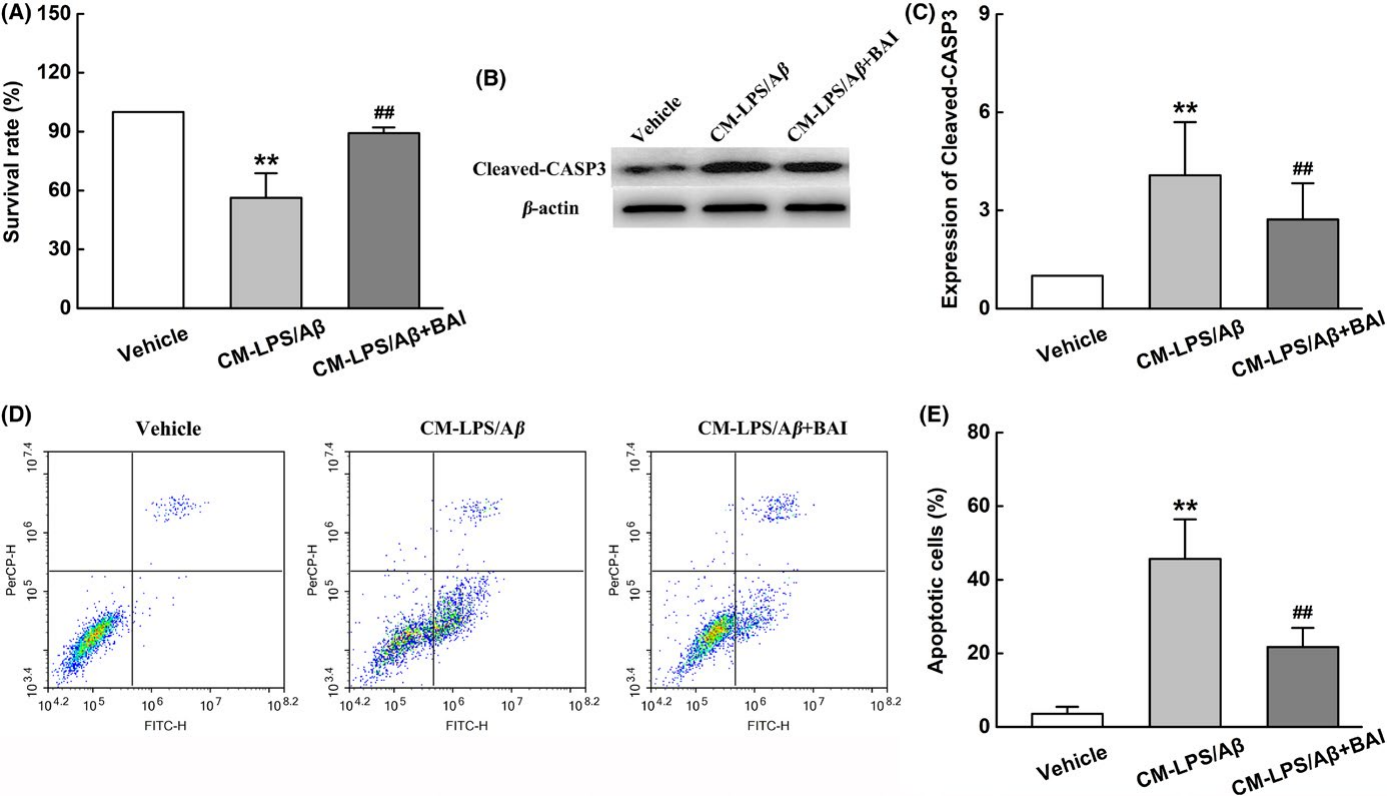
BAI reduced the damage to neuron treated with conditioned media from LPS/Aβ‐induced BV2 cells. (A) The viability of SH‐SY5Y cells assessed using the CCK‐8 method. (B) Representative immunoblots probed with antibodies against Cleaved‐CASP3 and β‐actin. (C) Quantification of the level of Cleaved‐CASP3 normalized to β‐actin. (D) The percentage of apoptotic SH‐SY5Y cells assessed using Annexin V/PI staining flow cytometry analysis. (E) The percentage of apoptotic cells analyzed using Annexin V/PI staining flow cytometry analysis. All data are presented as mean ± SD (n = 4). **P < 0.01, compared with Vehicle group; ##*P* < 0.01, compared with CM‐LPS/Aβ group

We determined the effects of BAI on microglia neurotoxicity and neuronal loss in LPS/Aβ‐induced BV2 cells. Accordingly, we employed a microglia‐conditioned media system to evaluate whether the alleviation of microglia neurotoxicity by BAI is involved in the survival of neural cells. The CM derived from LPS/Aβ‐induced BV2 microglia with or without BAI pretreatment was added to SH‐SY5Y cells. A CCK‐8 assay revealed that the conditioned media derived from BV2 cells pretreated with BAI increased the viability of SH‐SY5Y cells, when compared to cells in a vehicle group (Figure 7A). In agreement with this observation, a Western blot of Cleaved‐CASP3, a classic indicator of apoptosis, showed that LPS/Aβ‐induced CM stimulated obvious neuronal apoptosis, whereas BAI treatment significantly alleviated neuronal apoptosis (Figure 7B, C). Furthermore, an Annexin V/PI staining flow cytometry assay also indicated that the CM from BV2 cells pretreated with BAI significantly reduced apoptosis in SH‐SY5Y cells (Figure 7D, E). The above results demonstrated that the neuroprotective effect of BAI may involve the inhibition of the microglia‐mediated neuroinflammatory response.

### 3.8 BAI attenuated the activation of NLRP3 inflammasomes and TLR4/NF‐κB pathway in LPS/Aβ‐induced BV2 cells

**Figure 8:**
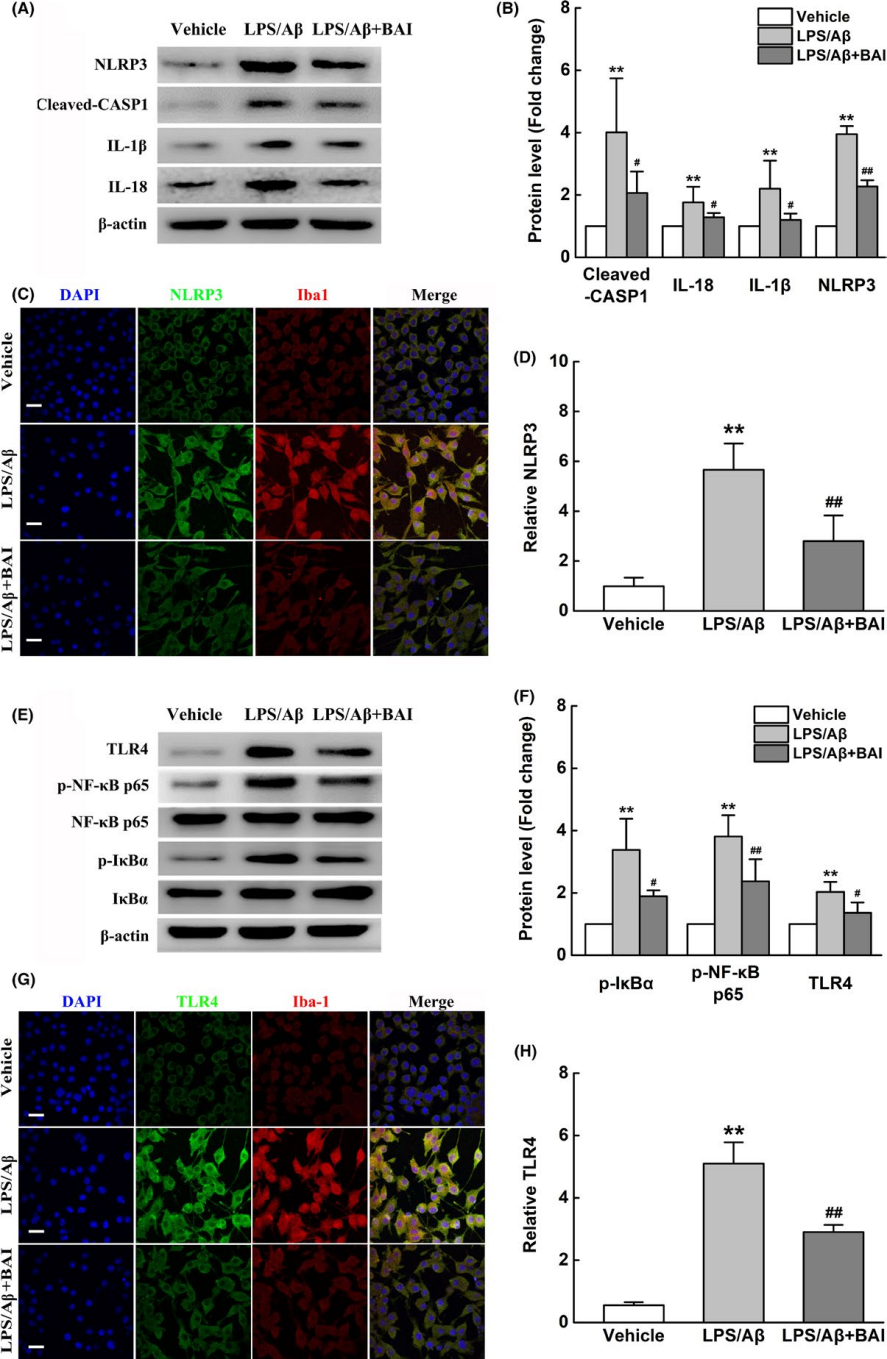
BAI treatment reduced the activation of NLRP3 inflammasomes and TLR4/NF‐κB signaling pathway in LPS/Aβ‐induced BV2 cells. (A) Representative immunoblots probed with antibodies against NLRP3, Cleaved‐CASP1, IL‐1β, IL‐18, and β‐actin. (B) Quantification of the levels of NLRP3, Cleaved‐CASP1, IL‐1β, and IL‐18 normalized to β‐actin. (C) Fluorescent Iba‐1 (red)/NLRP3 (green)/DAPI (blue) immunostaining in LPS/Aβ‐induced BV2 cells. Scale bar: 30 μm. (D) Quantification of NLRP3 immunofluorescent intensity. (E) Representative immunoblots probed with antibodies against TLR4, p‐NF‐κB p65, NF‐κB p65, p‐IκBα, IκBα, and β‐actin. (F) Quantification of the levels of TLR4, p‐NF‐κB p65, NF‐κB p65, p‐IκBα, and IκBα normalized to β‐actin. (G) Fluorescent Iba‐1 (red)/ TLR4 (green)/DAPI (blue) immunostaining in LPS/Aβ‐induced BV2 cells. Scale bar: 30 μm. (H) Quantification of TLR4 immunofluorescent intensity. All data are presented as mean ± SD (n = 4). **P < 0.01, compared with vehicle group; #*P* < 0.05, ##*P* < 0.01, compared with LPS/Aβ‐treated group

NLRP3 inflammasomes play a major role in transcriptional regulation of proinflammatory cytokines.^29^ To identify the mechanisms underlying the regulation of microglial activation by BAI, we examined the effects of BAI on NLRP3 inflammasome activation in LPS/Aβ‐induced BV2 cells. As expected, Western blot results showed that the markedly enhanced protein expression of NLRP3, Cleaved‐CASP1, IL‐1β, and IL‐18 induced by LPS/Aβ was significantly suppressed by BAI treatment (Figure 8A, B). In support of this, immunofluorescence also showed that BAI significantly inhibited NLRP3 protein expression (Figure 8C, D). Overall, these results indicated that the inhibitory inflammatory effects of BAI are mediated by suppression of the activation of NLRP3 inflammasomes in microglia.

Since it is well known that the TLR4/NF‐κB pathway plays an essential role in regulating NLRP3 inflammasome activation, we investigated the potential effects of BAI on the TLR4/NF‐κB pathway in microglia. Western blot analysis revealed that BAI significantly suppressed NF‐κB p65 nuclear translocation in response to LPS/Aβ, which was accompanied by inhibition of the LPS/Aβ‐induced increase in p‐IκBα and the decrease in IκBα protein expression (Figure 8E, F). In addition, we found that BAI significantly attenuated the LPS/Aβ‐stimulated increase in TLR4 protein levels in BV2 microglia (Figure 8G, H). Based on the above data, we concluded that BAI achieved its anti‐inflammation effects by inhibiting the activation of NLRP3 inflammasomes and blocking the TLR4/NF‐κB pathway in LPS/Aβ‐induced BV2 cells.

## 4 DISCUSSION

The main pathological features of AD are progressive loss of memory and deterioration of cognitive function. BAI, the major flavonoid glycoside responsible for the biological activity of *S. baicalensis,* has been hypothesized to possess neuroprotective effects.^30^ A previous study reported that BAI effectively improved memory deficits and reduced AD‐like pathological changes in Aβ‐injected ICR mice.^31^ Compared to an AD rat model, APP/PS1 mice, which overexpress the Swedish mutation of APP and contain a deletion of exon 9 in PS1, are more reliable and easily operable, and they serve as a powerful model for AD research. However, prior to the current study, the molecular mechanisms underlying the effects of BAI in APP/PS1 mice remained obscure. The first Aβ deposits form in the neocortex of APP/PS1 mice at 6 weeks of age. As the mice grow older, the Aβ deposits increase in size and number, and diffuse amyloid deposits begin to develop. At 8 months of age, intensive Aβ plaques surrounded by diffuse amyloid deposits cover almost the entire neocortex.^32^ Our previous studies found that 12‐month‐old APP/PS1 mice displayed robust Aβ plaque formation throughout the hippocampus and cortex as well as obvious cognitive deficits.^33^ Because the development of neuropathological changes in the hippocampal regions lags behind that in the cortex, our study focused on whether BAI could reduce neural apoptosis resulting from neuroinflammation in the hippocampus, which is the key region responsible for memory formation. Therefore, to ensure the occurrence of more serious injuries in the hippocampus, relatively elderly 14‐month‐old APP/PS1 mice were used for this study.

In consideration of future clinical applications, the dosage determination for BAI treatment was based on regulations regarding the maximum daily dosage of *Radix Scutellariae* (≤10.0 g per 70.0 kg of adult weight) and the minimum amount of BAI in *R. Scutellariae* (≥8.0%) given in the Chinese Pharmacopoeia (2015 Edition). Thus, the final intragastric dosage of BAI in our study was ascertained to be 103 mg/kg/day. Upon examining previously published articles, we found that in different rat or mouse models of dementia, the length of BAI treatment used to determine its effects in improving learning and memory deficits generally ranged from 14 to 21 days.^15^, ^31^, ^34^, ^35^ Additionally, in our previous studies, the length of memantine and (‐)‐epigallocatechin‐3‐gallate treatment used to determine their effects on AD‐like behavior changes in APP/PS1 mice was customarily 4 weeks.^20^, ^33^ Therefore, the 33‐day treatment length used in this study should be long enough to reveal any anti‐AD properties of BAI. Our behavioral data demonstrated for the first time that after 33 days of treatment, BAI could effectively alleviate spatial memory deficits and learning impairments in APP/PS1 mice, as assessed by PAT and MWM tests. In addition, BAI treatment did not significantly affect the locomotivity and frequencies of stand‐up during the motor function test. These findings are consistent with those of previous studies that raised concerns about the potential benefits of BAI in the treatment of AD. Consistent with the idea that BAI could lead to a reduction in Aβ deposition and recovery of cognitive function, we used a daily dose of BAI to assess its effects on Aβ deposition in APP/PS1 mice. Immunofluorescence results showed no significant differences between the APP/PS1 and BAI‐treated groups in terms of Aβ deposits in the hippocampus; however, the tendency toward decline in the BAI‐treated group was obvious. The results from a previous report also indicated that 30 days of BAI treatment (100 mg/kg) had no significant effects on the average Aβ load in the cortex of 12‐month‐old APP/PS1 mice.^36^ These results indicated that BAI did not reverse the degree of Aβ load in mature APP/PS1 mice. While Aβ accumulation may be a primary event in AD pathogenesis, microglial activation during neuroinflammation also plays an important role in disease progression. Activated microglia, characterized by branching processes and an enlarged cell shape, produce a wide spectrum of proinflammatory cytokines, such as IL‐1β, iNOS, IL‐18, and other mediators, which ultimately induce neuronal damage in the AD‐affected brain.^37^ Therefore, the inhibition of microglial activation is a key goal for the treatment of AD. Thus, we investigated whether BAI could suppress microglial activation and inhibit inflammatory factors. Immunological analysis found that APP/PS1 mice showed substantial microglial activation both in the vicinity and in areas surrounding Aβ deposition and that BAI treatment significantly attenuated microglial activation. Further study revealed that BAI significantly inhibited LPS/Aβ‐induced BV2 microglial activation, which is a finding that confirms an earlier observation, but at a much lower concentration (40 μmol/L) than that used in previous experiments.^36^ Meanwhile, in these activated microglia, significant increases in iNOS, IL‐1β, and IL‐18 levels were present as revealed using RT‐PCR and ELISA. BAI administration also suppressed the levels of iNOS, IL‐1β, and IL‐18 both in vitro and in vivo. These data provided new evidence that BAI is a promising natural compound that can be used to suppress microglial activation and neuroinflammation.

Neuronal apoptosis induced by neuroinflammation is recognized as the fate of neurodegenerative neurons during the development of AD, which exacerbates the resulting spatial and temporal deficits.^38^ Cleaved‐CASP3 has been identified as an apoptosis executioner in neuronal cells.^39^ Cleaved‐CASP3 activation can result in DNA condensation or fragmentation, which further contributes to mitochondrial dysfunction, and caspase cascade events, and ultimately leads to neuronal death. We tested the effects of BAI on apoptosis‐related proteins in vivo and in vitro during this study. The results indicated that Cleaved‐CASP3 was induced in SH‐SY5Y cells treated with CM from LPS/Aβ‐stimulated BV2 cells and the hippocampus of APP/PS1 mice and that BAI effectively reduced the expression of Cleaved‐CASP3. In addition, the number of Cleaved‐CASP3‐positive neurons in the hippocampus of APP/PS1 mice was markedly reduced in the BAI‐treated group, as indicated by histological analysis. Furthermore, Annexin V/PI staining flow cytometry assays also showed that BAI protected against the effects of conditioned media derived from LPS/Aβ treatment in SH‐SY5Y cells. On the basis of these results, BAI had protective effects against inflammation‐induced neuronal apoptosis in SH‐SY5Y cells and APP/PS1 mouse brains.

A better understanding of the mechanisms underlying the effects of BAI on neuroinflammation would provide novel insights that could lead to the development of effective anti‐AD drugs. The NLRP3 inflammasome, a multiprotein complex that includes NLRP3, ASC, and CASP1, is a key signaling mediator in Aβ‐triggered microglial inflammatory activation.^40^ The NLRP3 inflammasome is known to cleave inactive pro‐IL‐1β and pro‐IL‐18 into mature, active IL‐1β and Il‐18, which subsequently allows for the secretions of these mature forms from microglial cells.^41^ Due to the release of proinflammatory cytokines, the numerous neuroinflammation processes further exacerbate the pathological processes that cause AD.^42^ Evidence suggests that deletion of NLRP3 significantly attenuates neuroinflammation and improves AD‐like pathology in APP/PS1 mice.^43^ A previous study has reported that flavocoxid, a BAI/catechin formulation, reduced the activation of NLRP3 and IL‐1β production in the cortex of 3xTg‐AD mice.^44^ In contrast, it has still not been demonstrated that BAI, as a mono‐compound, can inhibit NLRP3 inflammasome in any AD model. In our study, we first found that the protein expressions of NLRP3 and Cleaved‐CASP1 were remarkably increased in both APP/PS1 mice and LPS/Aβ‐induced BV2 cells. We noted that pretreatment with BAI effectively decreased the levels of IL‐1β and IL‐18, which was due to the downregulation of NLRP3 protein expression and inhibition of CASP1 activity. These findings, as mentioned above, indicated that the anti‐inflammation effects of BAI may be related to its inhibition of NLRP3 inflammasome activation. Furthermore, the signaling mechanism leading to NLRP3 inflammasome activation still requires clarification.

In the CNS, TLR4 is widely expressed on microglial surfaces and is involved in recognizing Aβ.^45^ Several studies have demonstrated that TLR4 plays an important role in the regulation of inflammation. Following binding with Aβ, TLR4 can promote NF‐κB complex activation and downstream events that participate in the transcriptional expression of NLRP3 and pro‐IL‐β.^27^, ^46^ The NF‐κB complex is a homodimer or heterodimer of proteins that consists of subunits p50 and p65. Under normal conditions, NF‐κB p50 and p65 dimers are maintained in an inactive state in the cytoplasm in complexes with IκB family inhibitor proteins. TLR4‐initiated signaling mediates the phosphorylation and degradation of IκB, which allows the NF‐κB p65 subunit to shuttle into the nucleus, where it binds with a specific DNA consensus sequence, which results in the enhancement of inflammation‐related protein transcription.^47^ Therefore, we used Western blot and immunofluorescence assays to determine whether BAI affects TLR4/NF‐κB signaling both in vivo and in vitro. We found that in APP/PS1 mice and LPS/Aβ‐treated BV2 cells, TLR4 was activated, as indicated by its upregulation, and contributed to downstream activation of NF‐κB signaling that was characterized by enhanced phosphorylation of IκBα and NF‐κB p65 expression. In contrast, BAI significantly inhibited the upregulated expression of TLR4, suppressed the phosphorylation and degradation of IκBα, and inhibited the subsequent nuclear translocation of NF‐κB p65. Based on these findings, we suggested that the effects of BAI against NLRP3 inflammasome activation are mediated via downregulation of TLR4/NF‐κB signaling.

Overall, the data presented in this article suggest that BAI could rescue cognitive impairment, inhibit microglial activation, decrease neuroinflammation, and alleviate neuronal loss. Moreover, the mechanisms underlying the anti‐inflammation effects of BAI may involve the suppression of NLRP3 inflammasomes and blockage of the TLR4/NF‐κB signaling pathway. These findings provide a new basis for the beneficial effects of BAI administration during AD development.

## CONFLICTS OF INTEREST

The authors declare no competing financial interests.
